# Sensory Ataxia Mimicking Diabetic Neuropathy in a Long-Term Metformin User: A Case Report From Korea

**DOI:** 10.7759/cureus.110704

**Published:** 2026-06-11

**Authors:** Joomong Park

**Affiliations:** 1 Medicine, Sacheon City Public Health Center, Sacheon, KOR

**Keywords:** cobalamin deficiency, diabetic neuropathies, empirical treatment, metformin, primary health care, sensory ataxia, subacute combined degeneration, vitamin b12 deficiency

## Abstract

A 66-year-old man with long-standing diabetes and prolonged metformin exposure presented with a 10-year history of distal paresthesia and progressive imbalance, previously attributed to diabetic neuropathy. Bedside examination demonstrated a positive Romberg sign, impaired distal joint position sense, and reduced vibration sense, suggesting sensory ataxia with posterior column involvement. Mild macrocytosis without anemia was identified. In a resource-limited primary care setting where confirmatory testing was not immediately available, empirical methylcobalamin was initiated. The patient subsequently reported resolution of paresthesia and substantial improvement in gait stability. This case highlights the importance of reassessing presumed diabetic neuropathy when bedside findings suggest an alternative and potentially reversible diagnosis.

## Introduction

Peripheral neuropathy in patients with diabetes is often attributed to diabetic neuropathy; however, alternative and potentially reversible causes may be overlooked [1]. Vitamin B12 deficiency is a well-recognized but frequently underdiagnosed cause of neurological dysfunction, particularly in primary care settings where access to specialized investigations may be limited [2]. Vitamin B12 deficiency may present with gait instability, proprioceptive deficits, and features of posterior column dysfunction, which may be misinterpreted as diabetic neuropathy [3,4]. Nutritional deficiency, malabsorption, and medication-related factors remain important causes of vitamin B12 deficiency in clinical practice [5]. Early recognition is critical, as neurological deficits may be reversible with timely treatment.

## Case presentation

A 66-year-old man with a history of hypertension, diabetes mellitus (approximately 30 years), and glaucoma presented to a primary care clinic for routine medication refill. The patient reported a long-standing history (approximately 10 years) of intermittent distal paresthesia in the hands and feet, accompanied by episodes of imbalance. The paresthesia was described as bilateral tingling and numbness in a stocking-glove distribution without radicular pain. The gait disturbance was most noticeable when climbing stairs or walking, and was often pointed out by others, as the patient did not perceive it clearly. The patient had been on long-term metformin therapy for approximately 30 years as part of diabetes management, which is a known risk factor for vitamin B12 deficiency [6].

Despite long-standing symptoms, the patient had not previously undergone evaluation for reversible causes of neurological dysfunction. There was no history of acute focal neurological deficits. Neurological examination revealed mild gait instability with cautious turning and difficulty during tandem walking. Gait was broad-based but not spastic. Muscle bulk and tone were preserved. Strength was 5/5 in bilateral hip flexion, knee extension, ankle dorsiflexion, and plantar flexion. Deep tendon reflexes were symmetric in the upper limbs, reduced at the ankles, and preserved at the knees. Plantar responses were flexor bilaterally. Sensory examination demonstrated reduced vibration sense at the great toes and impaired joint position sense distally in both lower extremities. Light touch was relatively preserved. The Romberg sign was positive. No cranial nerve deficit or cerebellar dysmetria was observed. There was no known history of diabetic nephropathy or foot ulceration. Ophthalmic history was significant for glaucoma; detailed retinopathy status was not immediately available at the visit. A formal assessment of microvascular complications was not completed at this encounter. Given the 30-year duration of diabetes, the possibility of diabetic neuropathy as a contributing or concurrent process was not excluded; notably, neuropathy may develop across a spectrum of dysglycemia, including prediabetes.

Laboratory investigations included complete blood count, renal function, liver function, and hemoglobin A1c (HbA1c). Laboratory results are summarized in Table 1. Notable findings included borderline macrocytosis (mean corpuscular volume (MCV) 94.7 fL, reference range 80-94 fL) and well-controlled glycemia (HbA1c 6.4%). The presence of borderline macrocytosis in the context of neurological symptoms raised suspicion for a possible vitamin B12 deficiency [2]. Due to limitations in diagnostic resources in the primary care setting, serum vitamin B12 levels and neuroimaging were not immediately available. Formal quantitative sensory testing was similarly not accessible at this facility; however, bedside assessment of vibration sense, joint position sense, and light touch was performed using standard clinical tools.

**Table 1:** Laboratory results at presentation Reference ranges are shown in parentheses. HbA1c, hemoglobin A1c; MCV, mean corpuscular volume; WBC, white blood cell count

Parameter	Result	Reference Range
WBC	8.08 × 10³/µL	4.0-10.0 × 10³/µL
Hemoglobin	14.4 g/dL	13.0-17.0 g/dL
Platelet count	258 × 10³/µL	150-400 × 10³/µL
MCV	94.7 fL	80-94 fL
Creatinine	1.03 mg/dL	0.70-1.20 mg/dL
HbA1c	6.40%	<5.7% normal; 5.7-6.4% prediabetes range

**Figure 1:**
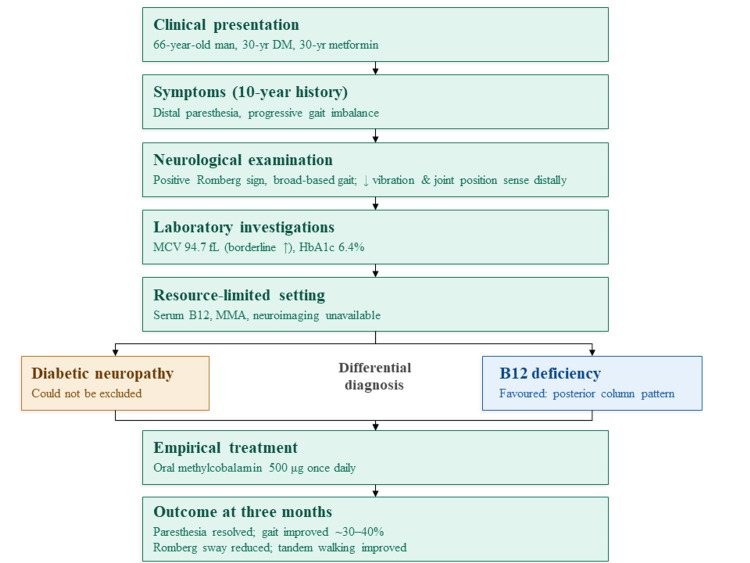
Diagnostic and management flowchart The posterior column pattern of sensory ataxia, disproportionate to typical diabetic neuropathy, guided clinical suspicion toward vitamin B12 deficiency in the absence of confirmatory biochemical testing. B12, vitamin B12; HbA1c, hemoglobin A1c; MCV, mean corpuscular volume; MMA, methylmalonic acid

Although the current HbA1c was 6.4%, diabetic neuropathy could not be excluded solely on this basis, as neuropathy may reflect cumulative historical glycemic burden. Historical longitudinal glycemic data were not fully available in this primary care encounter. However, the prominent sensory ataxia, proprioceptive impairment, and gait imbalance were considered disproportionate to typical distal symmetric diabetic polyneuropathy alone, prompting consideration of alternative or concurrent pathology. The diagnostic approach and clinical course are summarized in Figure 1.

Differential diagnoses included distal symmetric diabetic polyneuropathy, vitamin B12 deficiency-related myeloneuropathy with sensory ataxia, cervical spondylotic myelopathy, lumbar spinal stenosis, cerebellar or vestibular disorder, and alcohol-related neuropathy.

Oral methylcobalamin 500 µg once daily was initiated based on clinical suspicion of deficiency-related neurological dysfunction, a commonly used and low-risk regimen in outpatient practice. No treatment-related adverse effects were reported during follow-up.

The patient was also advised on lifestyle modifications, including cessation of alcohol and caffeine.

At follow-up, approximately three months after initiation of methylcobalamin 500 µg daily, the patient reported significant improvement in neurological symptoms. Distal paresthesia had resolved, gait instability had improved to approximately 30% to 40% of baseline severity, and no further noticeable imbalance during walking was reported. Tandem walking and turning were clinically smoother on repeat bedside assessment, and Romberg sway was less pronounced, although formal quantitative gait or sensory testing was not available.

## Discussion

The principal educational value of this case is not the use of empirical methylcobalamin itself, which is common clinical practice, but the recognition that long-standing neurological symptoms labelled as diabetic neuropathy may instead reflect a treatable sensory ataxia syndrome. In patients with diabetes, diagnostic anchoring to diabetic neuropathy may delay reconsideration of alternative causes, particularly when symptoms evolve gradually over many years.

This case highlights the diagnostic challenge of distinguishing diabetic neuropathy from other causes of neurological dysfunction in primary care. While diabetic neuropathy is common, prominent gait imbalance with proprioceptive loss should prompt consideration of additional or alternative pathology, particularly when symptoms appear disproportionate to distal sensory complaints [7]. Notably, the patient had experienced symptoms for nearly a decade without evaluation for reversible causes, highlighting a potential gap in the assessment of neurological symptoms in primary care settings. Previous reports have described vitamin B12 deficiency-related neurological dysfunction associated with long-term metformin use, often presenting with gait disturbance and sensory abnormalities [4,6]. Compared with these cases, the present case is notable for early or partial neurological involvement identified in a primary care setting before progression to classical subacute combined degeneration. Neuropathy may occur across a spectrum of dysglycemia, including prediabetes and overt diabetes. This does not exclude diabetic neuropathy as a contributing factor but suggests that the examination findings warranted consideration of an additional or alternative process.

The presence of gait disturbance, particularly when recognized by others rather than the patient, suggests impaired proprioception and possible posterior column dysfunction [3,4]. This pattern is more consistent with vitamin B12 deficiency-related neurological involvement than with typical diabetic peripheral neuropathy, which usually presents with distal sensory symptoms without significant imbalance in early stages. Vitamin B12 deficiency can affect both the peripheral nerves and the spinal cord, resulting in overlapping clinical features [3,4]. In this case, distal paresthesia may reflect peripheral nerve involvement, whereas gait instability suggests concomitant involvement of the dorsal columns.

Although the findings do not fulfil the classical criteria for subacute combined degeneration, the combination of gait instability, long-standing paresthesia, and clinical improvement following methylcobalamin supplementation is suggestive of early or partial involvement of the posterior columns.

In this case, mild macrocytosis provided an important clinical clue despite the absence of overt anemia. Vitamin B12 deficiency may present with neurological symptoms even in the absence of hematological abnormalities [2,8,9]. Long-term metformin use has been associated with vitamin B12 deficiency and may contribute to under-recognized neurological manifestations in patients with diabetes [6,10]. Monitoring of vitamin B12 levels in long-term metformin users has been recommended in clinical guidelines [11].

Due to limitations in diagnostic resources, a therapeutic trial of methylcobalamin was initiated without biochemical confirmation. The substantial clinical improvement following supplementation, together with the overall clinical pattern, supports a possible deficiency-related neurological process. However, definitive confirmation of vitamin B12 deficiency was not available because serum vitamin B12 testing and additional confirmatory investigations could not be performed. Nevertheless, the resolution of paresthesia and functional gait improvement were clinically meaningful to the patient.

A limitation of this case is the lack of direct measurement of serum vitamin B12 levels, the absence of neuroimaging, incomplete access to longitudinal glycemic records, and the absence of formal post-treatment neurological testing. However, in resource-limited primary care settings, clinical reasoning combined with therapeutic response may still provide valuable diagnostic insight.

The relatively preserved glycemic control and limited documented microvascular burden support consideration of alternative diagnoses when neurological findings are disproportionate to the degree of glycemic burden, although the absence of known nephropathy or foot ulceration does not exclude diabetic neuropathy.

## Conclusions

This case emphasizes that gait imbalance, a positive Romberg sign, or impaired proprioception in a patient with diabetes should prompt reconsideration beyond diabetic neuropathy alone. Clinicians should avoid prematurely attributing chronic neurological symptoms solely to diabetes and should consider reversible causes such as vitamin B12 deficiency, particularly in long-term metformin users.

In resource-limited primary care settings, a structured bedside neurological assessment may be sufficient to raise clinical suspicion for cobalamin deficiency. When biochemical confirmation is unavailable, a carefully monitored therapeutic trial with methylcobalamin remains a reasonable approach, and clinical response may serve as supporting diagnostic evidence. Nevertheless, clinical improvement should not be considered definitive proof of vitamin B12 deficiency, and biochemical confirmation should be obtained whenever feasible.
